# *Plasmodium vivax* Reticulocyte Binding Proteins Are Key Targets of Naturally Acquired Immunity in Young Papua New Guinean Children

**DOI:** 10.1371/journal.pntd.0005014

**Published:** 2016-09-27

**Authors:** Camila T. França, Wen-Qiang He, Jakub Gruszczyk, Nicholas T. Y. Lim, Enmoore Lin, Benson Kiniboro, Peter M. Siba, Wai-Hong Tham, Ivo Mueller

**Affiliations:** 1 Population Health and Immunity Division, Walter and Eliza Hall Institute of Medical Research, Melbourne, Australia; 2 Department of Medical Biology, University of Melbourne, Melbourne, Australia; 3 Infection and Immunity Division, Walter and Eliza Hall Institute of Medical Research, Melbourne, Australia; 4 Malaria Immuno-Epidemiology Unit, PNG Institute of Medical Research, Madang, Papua New Guinea; 5 Malaria Parasites and Hosts Unit, Department of Parasites and Insect Vectors, Pasteur Institute, Paris, France; 6 Barcelona Institute of Global Health (ISGLOBAL), Barcelona, Spain; Academic Medical Centre, NETHERLANDS

## Abstract

**Background:**

Major gaps in our understanding of *Plasmodium vivax* biology and the acquisition of immunity to this parasite hinder vaccine development. *P*. *vivax* merozoites exclusively invade reticulocytes, making parasite proteins that mediate reticulocyte binding and/or invasion potential key vaccine or drug targets. While protein interactions that mediate invasion are still poorly understood, the *P*. *vivax* Reticulocyte-Binding Protein family (PvRBP) is thought to be involved in *P*. *vivax* restricted host-cell selectivity.

**Methodology/Principal findings:**

We assessed the binding specificity of five members of the PvRBP family (PvRBP1a, PvRBP1b, PvRBP2a, PvRBP2b, PvRBP2-P2 and a non-binding fragment of PvRBP2c) to normocytes or reticulocytes. PvRBP2b was identified as the only reticulocyte-specific binder (P<0.001), whereas the others preferentially bound to normocytes (PvRBP1a/b P≤0.034), or showed comparable binding to both (PvRBP2a/2-P2, P = 0.38). Furthermore, we measured levels of total and IgG subclasses 1, 2, 3 and 4 to the six PvRBPs in a cohort of young Papua New Guinean children, and assessed their relationship with prospective risk of *P*. *vivax* malaria. Children had substantial, highly correlated (rho = 0.49–0.82, P<0.001) antibody levels to all six PvRBPs, with dominant IgG1 and IgG3 subclasses. Both total IgG (Incidence Rate Ratio [IRR] 0.63–0.73, P = 0.008–0.041) and IgG1 (IRR 0.56–0.69, P = 0.001–0.035) to PvRBP2b and PvRBP1a were strongly associated with reduced risk of vivax-malaria, independently of age and exposure.

**Conclusion/Significance:**

These results demonstrate a diversity of erythrocyte-binding phenotypes of PvRBPs, indicating binding to both reticulocyte-specific and normocyte-specific ligands. Our findings provide further insights into the naturally acquired immunity to *P*. *vivax* and highlight the importance of PvRBP proteins as targets of naturally acquired humoral immunity. In-depth studies of the role of PvRBPs in *P*. *vivax* invasion and functional validation of the role of anti-PvRBP antibodies in clinical immunity against *P*. *vivax* are now required to confirm the potential of the reticulocyte-binding PvRBP2b and PvRBP1a as vaccine candidate antigens.

## Author Summary

In parallel with the tremendous reduction in malaria burden, *Plasmodium vivax (Pv)* is now the predominant malaria species in the Asia-Pacific and Americas. *Pv* can only invade young erythrocytes (reticulocytes) and this restriction is thought to involve the Reticulocyte-Binding Protein family (PvRBP). Given their predicted role, PvRBPs are potentially interesting vaccine targets. However, the acquisition of immunity to *Pv* in general (PvRBPs in particular) is poorly understood, hindering vaccine development. Here, we show that out of five PvRBPs, only one (PvRBP2b) binds exclusively to reticulocytes. Furthermore, we measured antibody levels to all six PvRBPs in a cohort of young Papua New Guinean children, assessing the relationship between antibodies to PvRBPs and risk of malaria disease. Both total and specific antibody subclass levels (IgG1 and IgG3) to the reticulocyte-specific binder PvRBP2b, and the non-specific binder PvRBP1a were strongly associated with lower risk of clinical disease. Our findings indicate a diversity of roles of PvRBPs in erythrocyte invasion and highlight their importance as targets of the naturally acquired immunity to *Pv*. Functional studies of the role of PvRBPs in reticulocyte invasion will be required to fully understand the potential of PvRBP1a and PvRBP2b as vaccine candidates.

## Introduction

The two major malaria parasites, *Plasmodium falciparum* and *Plasmodium vivax*, differ in their ability to invade human erythrocytes. While *P*. *falciparum* invades both mature (normocytes) and young erythrocytes (reticulocytes), *P*. *vivax* can only invade the latter [1]. This differential specificity is believed to be mediated by distinct ligand-receptor interactions, though the exact mechanisms remain to be elucidated [1]. For *P*. *falciparum*, the merozoite invasion of erythrocytes is a multistep process [2] mediated by the binding of the erythrocyte binding-like (EBL) and reticulocyte binding-like (PfRh) protein families to receptors on the surface of the host cell [3]. For *P*. *vivax*, the only ligand-receptor interaction identified to date is between the Duffy binding protein (PvDBP) and the Duffy antigen receptor for chemokines (DARC) [4]. The recently identified *P*. *vivax* erythrocyte-binding protein (PvEBP) also shares a Duffy binding-like domain [5]. However, the presence of DARC in both normocytes and reticulocytes does not explain the restricted host-cell selectivity of *P*. *vivax*. The recent observation that *P*. *vivax* can invade Duffy-negative cells also indicates the existence of alternative pathways of invasion [6, 7].

The *P*. *vivax* reticulocyte binding brotein family (PvRBP) is composed of 11 members [5, 8, 9], and although their precise roles remain largely unknown, their homology to the much better characterized PfRh protein family suggests that they may be important invasion ligands [3]. Members of the PvRBP family have been implicated in erythrocyte binding, and in some cases in reticulocyte recognition [8, 10]. Variation in expression of PvRBPs genes in different parasite isolates have been described, suggesting that these genes may be redundant in function [11]. The relatively high degree of polymorphism observed in the genes encoding PvRBPs also indicates that they are important for parasite survival and may be under immune selection [10, 12, 13]. Collectively, this suggests that the understudied PvRBP family may be of key importance for *P*. *vivax* invasion, and like their better studied *P*. *falciparum* homologues, potential targets for a vaccine targeting blood stage infections [14].

Antibodies to several *P*. *vivax* merozoite proteins have shown associations with reduced risk of vivax-malaria in naturally exposed individuals [15]. Among them, antibodies to PvDBPs have been target of extensive study [15]. While PvDBP is a promising vaccine candidate, several challenges to vaccine development remain, including the presence of highly polymorphic, immuno-dominant epitopes in the DARC-binding region II, and the need to elicit high titers to achieve strain-transcending blocking [14]. It is therefore likely that PvDBP would need to be combined with further antigens targeting alternative invasion ligands. Members of the PvRBP family are recognized by antibodies from vivax-positive patients [11, 16], and populations living in endemic areas [17, 18]. Yet, an in-depth characterization of the immune responses to these proteins, as well as the role of antibodies to PvRBPs in the acquisition of immunity to malaria is lacking.

In this study, we have assessed the erythrocyte-binding profiles of five members of the PvRBP family and their specificity to normocytes or reticulocytes, identifying a member of the PvRBP family that exclusively binds to reticulocytes. Furthermore, we measured levels of total and IgG subclasses to these five PvRBPs and a non- erythrocyte binding protein fragment of a sixth PvRBP in a cohort of young Papua New Guinean (PNG) children with well-characterized differences in exposure. We identified an association between reduced risk of vivax-malaria and antibodies to two of the PvRBPs, including the reticulocyte-specific binder. Our results provide important insights into the acquisition of immunity to PvRBPs in young children, highlighting this protein family as an interesting target to be further evaluated for their potential as *P*. *vivax* vaccine antigens.

## Methods

### PvRBPs expression and purification

Proteins included in this study were PvRBP1a (amino acids [aa] 160–1170), PvRBP1b (aa 140–1275), PvRBP2a (aa 160–1135), PvRBP2b (aa 161–1454), PvRBP2cNB (aa 501–1300) and PvRBP2-P2 (aa 161–641) Their expression and purification have been described in details elsewhere [10, 11]. Despite several attempts, we were unsuccessful in expressing recombinant PvRBP2c that includes the conserved erythrocyte-binding domain in *E*. *coli* using both native and refolding methods. Thus, the PvRBP2cNB fragment included in this study does not contain the erythrocyte-binding domain, which encompasses residues 128 to 429. An SDS-PAGE of PvRBP2-P2 recombinant protein is shown in S1 Fig; the purity and stability of the remaining PvRBPs have been verified and presented in a previous publication [11].

### Polyclonal rabbit anti-PvRBP antibody production

Antibody production was performed at the Walter and Eliza Hall Institute Monoclonal Antibody Facility as previously described [10].

### Screening of rabbit anti-PvRBP IgG by enzyme-linked immunosorbent assay (ELISA)

96-well flat-bottomed plates (Maxisorp, Nunc) were coated with each of the 6 PvRBPs (65 nM/well) in individual wells and incubated for two hours. For 65 nM of protein in 100 μL, we added 0.8 μg, 0.9 μg, 0.7 μg, 1 μg, 0.6 μg and 0.4 μg for PvRBP1a, PvRBP1b, PvRBP2a, PvRBP2b, PvRBP2cNB and PvRBP2_P2 respectively. Plates were blocked with 5% skim milk/0.1% Tween-20 for one hour. After washing, specific anti-PvRBP polyclonal antibodies (1 mg/mL stock) were added at halving serial dilutions (from 1:2000 to 1:64000) for one hour. Plates were washed three times before the addition of HRP-goat anti-rabbit secondary antibodies (1:2000 dilution) for one hour. Azino-bis-3-ethylbenthiazoline-6-sulfonic acid (ABTS liquid substrate; Sigma-Aldrich) was used to detect HRP activity. 1% SDS was used to stop the reaction and absorbance was measured at 405 nm. All experiments were performed at room temperature. All washes were done in PBS/0.1% Tween-20, and dilutions of antibodies in 0.5% skim milk/0.1% Tween-20. Samples were tested in duplicates.

### Flow cytometry-based erythrocyte-binding assay

Reticulocytes were enriched from whole blood and the erythrocyte-binding assays performed as described previously [10]. Binding of PvRBPs was detected using 0.025 mg/mL of corresponding anti-PvRBP rabbit IgG.

### Study population

Antibody reactivity to the 6 PvRBPs in naturally-exposed individuals was assessed in samples from a longitudinal cohort of 264 children (1–3 years old) undertaken in Ilaita, East Sepik Province, PNG [19]. Children were enrolled between March-September 2006, and followed for up to 16 months. Blood samples were collected every eight weeks and at episodes of febrile illness. All *P*. *vivax* infections were genotyped, allowing the determination of the incidence of genetically distinct blood-stage infections acquired during follow-up (i.e. the molecular force of blood-stage infections, molFOB) [20]. Samples collected at enrolment from 224 children that completed follow-up were included in the present study (median age 1.7, inter-quartile range [IQR] 1.3–2.5).

### Protein conjugation and antibody measurement

To measure antibody levels in the cohort of PNG children, purified proteins were conjugated to Luminex Microplex microspheres (Luminex Corp.) as described elsewhere [21, 22], using the following concentrations per 2.5 x 10^6^ beads: PvRBP1a = 3 μg/mL; PvRBP1b = 11.4 μg/mL; PvRBP2a = 6.7 μg/mL; PvRBP2b = 0.2 μg/mL; PvRBP2cNB = 0.8 μg/mL; PvRBP2-P2 = 5.4 μg/mL.

Bead-array assays were performed as previously described [23]. Plasma samples were diluted 1:50 in PBS, and secondary antibody donkey F(ab’)2 anti-human IgG Fc R-PE (1 mg/ mL, Jackson Immunoresearch); mouse anti-human IgG1 hinge-PE (0.1 mg/ mL, clone 4E3, Southern Biotech); IgG2 Fc-PE (0.1 mg/ mL, clone HP6002, Southern Biotech); IgG3 hinge-PE (0.1 mg/ mL clone HP6050, Southern Biotech); or IgG4 Fc-PE (0.1 mg/ mL, clone HP6025, Southern Biotech) diluted 1:100 in PBS was added to detect total, IgG1, IgG2, IgG3 or IgG4 respectively. A dilution series of a pool made of serum collected as part of an earlier study with immune adults living in different villages of high malaria transmission in East Sepik Province, PNG, was included on each plate as positive controls.

### Statistical analysis

To correct plate-to-plate variations, the dilutions of the PNG adult pool were fitted as plate-specific standard curves using a 5-parameter logistic regression model [22, 24]. For each plate, median fluorescence intensity (MFI) values were interpolated into relative antibody units based on the parameters estimated from the plate’s standard curve.

Associations with age, exposure and correlations between antibody levels of different subclasses and/or different antigens were determined using Spearman’s rank correlation, and differences by infection status using Mann-Whitney U tests. Generalized estimating equation (GEE) models with exchangeable correlation structure and semi-robust variance estimator were used to analyze the relationship between antibodies to PvRBPs and prospective risk of *P*. *vivax* episodes (defined as axillary temperature ≥ 37.5°C or history of fever in preceding 48 hours with a concurrent parasitaemia >500 *P*. *vivax* parasites/μl) over the 16 months of follow-up [22, 25]. For this, antibody levels were classified into tertiles (cut-off values are shown in Table 1), and analyses were done comparing the incidence rate ratio (IRR) of clinical malaria in those with medium and high versus low antibody levels. Children were considered at-risk from the first day after the blood sample for active follow-up was taken. For each child, the molFOB was calculated as the number of new blood-stage genetically distinct *P*. *vivax* clones acquired/year-at-risk, and square-root transformed for better fit [20]. Adjustments were made for seasonal trends, village of residency, age, and molFOB. In order to study the breadth of anti-PvRBP antibodies, for each antigen antibody levels stratified into tertiles were scored as 0 for low, 1 for medium and 2 for the high tertiles, respectively. Scores were then added up to reflect the breadth of anti-RBP antibodies, yielding a median score of 6 (IQR 2–9).

**Table 1:** Total and IgG subclass reactivity to PvRBPs in young Papua New Guinean children.

	PvRBP1a			PvRBP1b			PvRBP2a		PvRBP2b		PvRBP2cNB		PvRBP2-P2		
	Total IgG	IgG1	IgG3	Total IgG	IgG1	Total IgG	IgG1	IgG3	Total IgG	IgG1	Total IgG	IgG1	Total IgG	IgG1	IgG3
	Level* in children (% of adult levels)														
Median	0.0025 (12.3)	0.0051 (25.5)	0.0002 (0.85)	0.0019 (9.6)	0.0054 (27.0)	0.0035 (17.3)	0.0037 (18.5)	0.0002 (0.91)	0.0029 (14.4)	0.0067 (33.4)	0.0006 (3.0)	0.0011 (5.3)	0.0095 (47.4)	0.0057 (28.5)	0.0031 (15.5)
IQR	0.0010 (5.1)	0.0025 (12.5)	0.0001 (0.32)	0.0010 (5.0)	0.0033 (16.3)	0.0013 (6.7)	0.0011 (5.7)	0.0001 (0.46)	0.0013 (6.5)	0.0024 (11.8)	0.0003 (1.5)	0.0005 (2.7)	0.0041 (20.5)	0.0025 (12.7)	0.0012 (5.8)
	0.0053 (26.3)	0.0094 (46.8)	0.0006 (3.1)	0.0041 (20.4)	0.0105 (52.3)	0.0101 (50.4)	0.0132 (65.8)	0.0005 (2.8)	0.0059 (29.6)	0.0149 (74.6)	0.0015 (7.7)	0.0025 (12.7)	0.0181 (90.1)	0.0128 (64.1)	0.0079 (39.7)
Cut-off* low antibody levels	0.0013 (6.3)	0.0031 (15.6)	0.0001 (0.47)	0.0013 (6.4)	0.0039 (19.7)	0.0017 (8.6)	0.0017 (8.5)	0.0001 (0.56)	0.0019 (9.6)	0.0037 (18.5)	0.0004 (2.0)	0.0007 (3.4)	0.0056 (27.9)	0.0033 (16.8)	0.0016 (7.9)
Cut-off* medium antibody levels	0.0038 (19.0)	0.0077 (38.5)	0.0004 (2.0)	0.0029 (14.5)	0.0078 (39.2)	0.0074 (36.9)	0.0086 (43.1)	0.0003 (1.7)	0.0046 (23.0)	0.0119 (59.7)	0.0010 (5.10)	0.0018 (9.1)	0.0145 (72.3)	0.0107 (53.4)	0.0053 (26.4)
	Cumulative prevalence in children, n (%)														
% of adult levels (antibody cut-off value *)															
1% (≥ 0.0002)	221 (98.7)	224 (100.0)	106 (47.3)	222 (99.1)	224 (100.0)	222 (99.1)	218 (97.3)	107 (47.8)	223 (90.6)	223 (99.6)	190 (84.8)	214 (95.5)	224 (100.0)	223 (99.6)	215 (96.0)
5% (≥ 0.001)	169 (75.5)	216 (96.4)	34 (15.2)	169 (75.5)	218 (97.3)	182 (81.2)	174 (77.7)	23 (10.3)	181 (80.8)	211 (94.2)	77 (34.4)	115 (51.3)	223 (99.6)	209 (93.3)	175 (78.1)
10% (≥ 0.002)	127 (56.7)	184 (82.1)	16 (7.1)	109 (48.7)	208 (92.9)	139 (62.1)	139 (62.1)	9 (4.0)	147 (65.6)	177 (79.0)	45 (20.1)	67 (29.9)	212 (94.6)	186 (83.0)	142 (63.4)
25% (≥ 0.005)	57 (25.5)	114 (50.9)	3 (1.3)	49 (21.9)	123 (54.9)	94 (42.0)	104 (46.4)	0 (0.0)	65 (29.0)	135 (60.3)	27 (12.1)	33 (14.7)	158 (70.5)	119 (53.1)	77 (34.4)
50% (≥ 0.01)	28 (12.5)	46 (20.5)	1 (0.45)	17 (7.6)	57 (25.5)	56 (25.0)	67 (29.9)	0 (0.0)	22 (9.8)	83 (37.1)	15 (6.7)	19 (8.5)	105 (46.9)	80 (35.7)	46 (20.5)

Abbreviations: IQR = Interquartile range

*Values in arbitrary units. Values were interpolated from standard curves using a 5PL logistic regression model.

All analyses were performed using STATA version 12 (StataCorp) or R version 3.2.1 (http://cran.r-project.org).

### Ethics statement

Ethical clearance was obtained from the PNG Medical Research and Advisory Committee (MRAC 05.19), and the Walter and Eliza Hall Institute (HREC 07/07). Written informed consent was obtained from the parents or guardians all children participating in the cohort study.

## Results

### Erythrocyte-binding characteristics of PvRBPs

**Fig 1:**
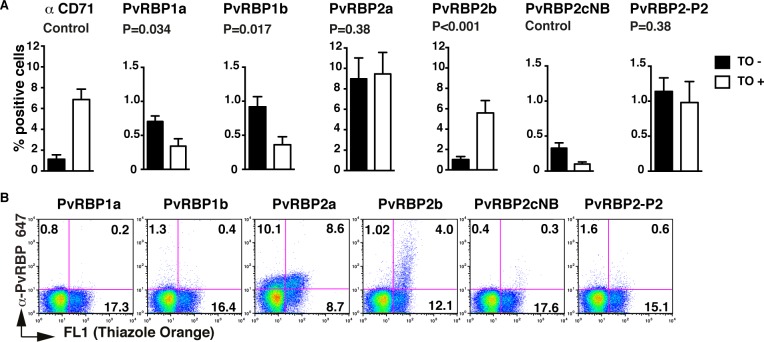
Erythrocyte-binding preferences of 6 PvRBPs. A: Bar charts showing the percentage of binding of CD71-PECy5 (control), PvRBP1a, PvRBP1b, PvRBP2a, PvRBP2b, PvRBP2cNB, PvRBP2-P2 to mature erythrocytes (not stained with thiazole orange, TO-) vs reticulocytes (stained with thiazole orange, TO+) populations. Error bars represent SEM of 7 or 9 independent repeats. B: Dot plots show the binding of six PvRBP proteins to an enriched reticulocyte population. Binding was detected using an anti-PvRBP rabbit IgG antibody followed by a secondary anti-rabbit Alexa 647 antibody.

Except for the PvRBP2cNB fragment, all PvRBPs proteins expressed encompass the conserved erythrocyte-binding domain [10]. As such, PvRBP2cNB binding serves as a control for background signal in this flow cytometry-based assay. Binding was significantly higher for all binding fragments except for PvRBP2b in normocytes (not stained with thiazole orange, TO-) and PvRBP1b in reticulocyes (stained with thiazole orange, TO +) (both P>0.05). Among the five binding PvRBPs tested we found three types of binding profiles: i) binding preferentially to normocytes: PvRBP1a (Fig 1, TO- vs. TO+: P = 0.034) and PvRBP1b (P = 0.017); ii) binding to both normocytes and reticulocytes: PvRBP2a (P = 0.38) and PvRBP2-P2 (P = 0.38); and iii) binding only to reticulocytes: PvRBP2b (P < 0.001).

### IgG to PvRBPs in young children

We assumed that the pooled serum from hyper-immune PNG adults represented the equilibrium antibody levels to all proteins achievable under life-long natural exposure. Therefore, by comparison with IgG levels observed in PNG children, we determined how many children have already achieved IgG levels that were >50%, >25% or >10% of the adult levels (Table 1).

Although, semi-immune, young PNG children were reactive to all six PvRBPs tested, there were differences in the immunogenicity of different proteins (Table 1; S2 Fig). Whereas, 47% and 95% of children had reached >50% and >10% of the hyper-immune adult levels for PvRBP2-P2, respectively, only 7% and 20% reached the same levels for antibodies targeting the non-red cell binding PvRBP2cNB fragment (P < 0.001). The other proteins were intermediately immunogenic, with antibodies to PvRBP2a more rapidly acquired than those to PvRBP2b, PvRBP1a and PvRBP1b (Table 1; S2 Fig). To each PvRBP, total IgG levels correlated moderate to strongly with IgG levels to the other PvRBPs (rho = 0.49–0.82, P < 0.001), with the strongest correlation between PvRBP1b and PvRBP2b (rho = 0.82) (S1 Table).

**Fig 2:**
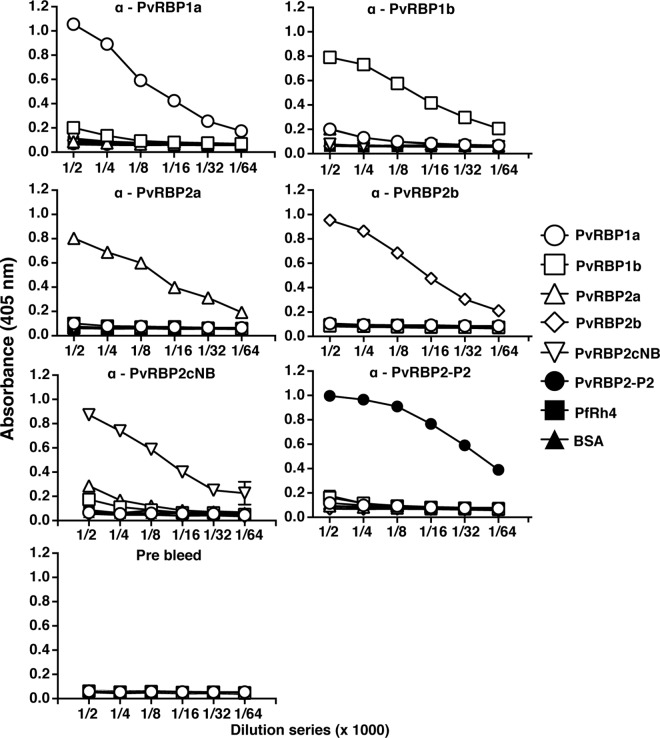
Detection of PvRBPs by rabbit anti-PvRBP polyclonal antibodies by ELISA. Microtiter wells were coated with each PvRBP per well as shown by symbols on the left. Solid lines show specific anti-PvRBPs polyclonal antibodies (top label) added to each plate in a dilution series. The optical density (OD) was measured at 405 nm. Mean OD values from duplicated wells and standard deviation are shown.

Rabbit polyclonal antibodies against the different PvRBP constructs showed strong recognition of the specific constructs but only limited cross-reactivity (Fig 2). The exception was antibodies raised against the PvRBP2cNB fragment, which showed low cross-reactivity with PvRBP1b and PvRBP2a. This indicated that the high correlations in naturally acquired total IgG levels are therefore likely to reflect co-acquisition rather than cross-reactivity (Fig 2).

### Influence of age, current and cumulative malaria exposure on IgG to PvRBPs

**Fig 3:**
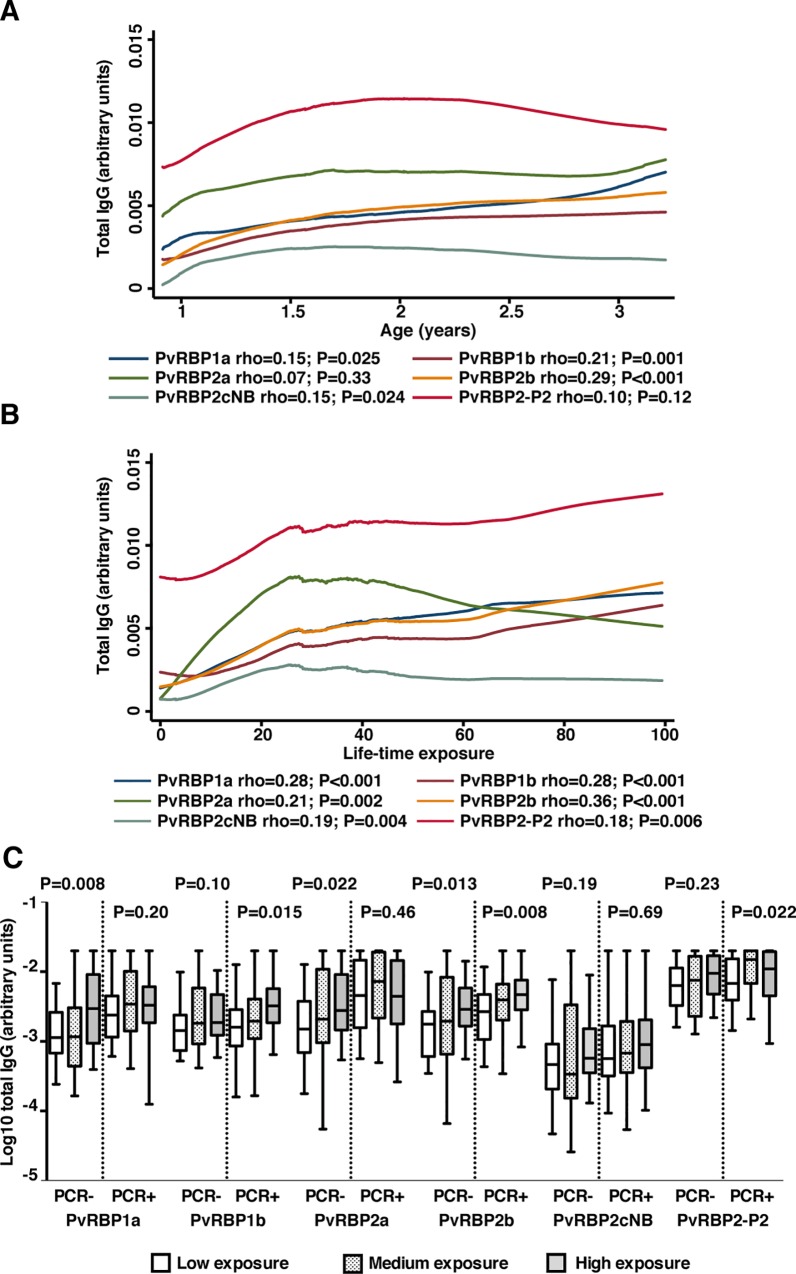
Association between total IgG to 6 PvRBPs age and exposure in 224 young Papua New Guinea children. A & B: Data are plotted as Lowess smoothed curves of total IgG levels (arbitrary units) by age and life-time exposure respectively. Life-time exposure was calculated as age multiplied by molFOB. Correlation coefficients (rho) and P-values from Spearman’s rank test. **C:** Boxplots show median total IgG levels (arbitrary units) and range (whiskers) by infection status detected by PCR. Clear boxes show low antibody levels, dotted boxes show medium antibody levels and grey boxes show high antibody levels. P-values from Kruskal-Wallis test. For all analysis, P < 0.05 were deemed significant.

While antibodies to PvRBP1a, PvRBP1b, PvRBP2b and PvRBP2cNB increased moderately with age (rho = 0.15–0.29, P < 0.001–0.025), no such association was found for the two most immunogenic proteins, PvRBP2a and PvRBP2-P2 (Fig 3A; S2 Table). Total IgG levels to all proteins except PvRBP1b were significantly higher in the 124 children (55.4%) that had a current, PCR-detectable *P*. *vivax* infection (P < 0.001–0.006) (S2 Table). To better understand the effect of age on the acquisition of antibodies to PvRBPs, we stratified children by the presence of infection at sample collection. After stratification, increase in total IgG to PvRBP1b, PvRBP2b, PvRBP2cNB and PvRBP2-P2 with age were stronger in children with current infection (rho = 0.19–0.33, P < 0.001–0.039) suggesting that antibodies to PvRBPs are strongly reflective of recent exposure (S2 Table).

Given the young age and large heterogeneity in exposure among children [20], age is not a good proxy for life-time exposure. As a better measure of life-time exposure to malaria, we therefore calculated the molFOB (described in methods) [20]. This estimated *P*. *vivax* life-time exposure was a substantially better predictor of antibody levels than age, and total IgG levels to all 6 PvRBPs significantly increased with increasing life-time exposure (rho = 0.18–0.36, P < 0.001–0.006) (Fig 3B; S2 Table). For PvRBP1a and PvRBP2a, the effect of life-time exposure to *P*. *vivax* was observed only in children free of infection at study start (P = 0.008–0.022), again indicating that the effect of recent infections on antibodies to PvRBPs is strong (Fig 3C; S2 Table).

The breadth of anti-PvRBP antibodies (described in methods) was higher in children with concurrent *P*. *vivax* infection (median in children free of infection = 4.5, IQR = 2–8 versus median in infected children = 7, IQR = 4–10; P < 0.001), and increased with increasing estimated life-time exposure (rho = 0.29, P < 0.001).

### IgG subclasses to PvRBPs

In hyper-immune PNG adults, four different patterns of IgG subclass reactivity to PvRBPs were observed i) predominant IgG1 with sub-dominant IgG3: PvRBP2-P2; ii) predominant IgG3 with sub-dominant IgG1: PvRBP1a; iii) predominant IgG1 with sub-dominants IgG2+IgG3: PvRBP2a, PvRPB2b and PvRBP2cNB; and iv) IgG1+IgG2+IgG3 with no obvious dominance: PvRBP1b. There were no detectable levels of IgG4 to any of the proteins (Table 1; S3 Fig).

In comparison to adults, young children had already acquired substantial IgG1 levels to all 6 PvRBPs. Apart from the less immunogenic PvRBP2cNB fragment, >20% and >62% of the children had reached >50% and >10% of the IgG1 levels seen in hyper-immune adults to the different PvRBPs. PvRBP1a, PvRBP2-P2 and PvRBP2a also had detectable levels of IgG3, but only PvRBP2-P2 had a similarly high prevalence of IgG3 as for IgG1 (20.5% and 63.4%, respectively) (Table 1; S3 Fig), indicating that IgG1 antibodies were acquired faster than IgG3. The predominance of IgG1 subclass is further highlighted by the generally stronger correlations of total IgG with IgG1 (rho = 0.91–0.94, P < 0.001) than IgG3 (rho = 0.55–0.71, P < 0.001) (S1 Table). Despite the narrow age group in the cohort, there was a weak indication of polarization towards IgG3 with increasing age to PvRBP1a (rho = -0.19, P = 0.005) and PvRBP2-P2 (rho = -0.16, P = 0.015), evidenced as a decrease in the IgG1/IgG3 ratio. Children had no detectable IgG2 or IgG4 to any of the proteins (S3 Fig).

IgG1 to all 6 PvRBPs (P ≤ 0.003) were higher in infected children, although only moderately to PvRBP1b and PvRBP2cNB (P = 0.06). Similarly, those with a current infection had higher IgG3 to PvRBP1a, PvRBP2a and PvRBP2-P2 (P ≤ 0.002) (S2 Table). For the PvRBPs with dominant IgG1 subclass, the effect of age and exposure mostly mimic that observed for total IgG (S2 Table). For PvRBP2-P2 and PvRBP1a IgG3, but not IgG1, still increased with age (rho = 0.19–0.21, P = 0.002–0.005) (S2 Table).

### IgG to PvRBPs and protection against symptomatic malaria

**Fig 4:**
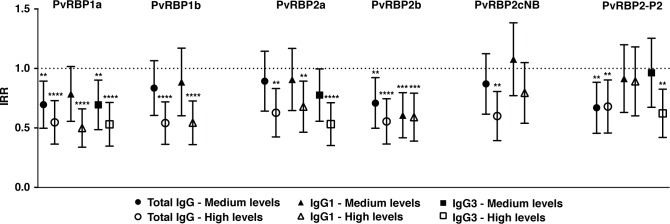
Association between total and IgG subclasses to 6 PvRBPs and protection against clinical malaria (density > 500/μL) in 224 young Papua New Guinean children. Data are plotted as exposure (molFOB), age, season and village of residency adjusted incidence rate ratios and 95% confidence intervals. Incidence rate ratios, 95% confidence intervals and P-values from GEE models. P < 0.05 were deemed significant. ****P < 0.001; ***P = 0.001; ** P > 0.001 to 0.01; *P > 0.01 to 0.05.

Over the 16 months follow-up of the PNG cohort, each child had an incidence rate of 1.25 (_95%_CI 1.08–1.45) *P*. *vivax* episodes/year at risk. Following adjustment for confounders, total IgG to all 6 PvRBPs tested were associated with protection against *P*. *vivax* malaria (IRR 0.52–0.69, P < 0.001–0.016) (Fig 4; S3 Table).

To further understand the contribution of antibodies to specific PvRBPs in the protective effect observed, we fitted a multivariate model to account for the fact that antibodies to the 6 PvRBPs were co-acquired and therefore highly correlated. In multivariate analysis, only total IgG to PvRBP1a (IRR_M_ 0.71, _95%_CI 0.53–0.94, P = 0.019; IRR_H_ 0.63, _95%_CI 0.44–0.88, P = 0.008), and PvRBP2b (IRR_M_ 0.73, _95%_CI 0.54–0.99, P = 0.041; IRR_H_ 0.63, _95%_CI 0.44–0.90, P = 0.011) remained associated with reduced risk of vivax-malaria, suggesting that antibodies to these two PvRBPs are important correlates of naturally-acquired protective immunity.

### IgG subclasses to PvRBPs and protection

After adjusting for confounders, both IgG1 and IgG3 to PvRBP1a (IgG1 IRR 0.48, P < 0.001; IgG3 IRR 0.51–0.67, P < 0.001–0.011) and PvRBP2a (IgG1 IRR 0.66, P = 0.010; IgG3 IRR 0.51, P < 0.001–0.011), and only IgG3 to PvRBP2-P2 (IRR 0.60, P = 0.002) were associated with reduced risk of vivax-malaria. IgG1 to PvRBP1b (IRR 0.52, P < 0.001) and PvRBP2b (IRR 057–0.59, P < 0.001) was also associated with protection. No association was found for the non-binding PvRBP2cNB fragment, although it was observed for total IgG levels in univariate analysis (Fig 4; S3 Table).

In a model combining both IgG1 and IgG3 levels to a given antigen, both IgG1 and IgG3 remained significantly associated with protection for PvRBP1a (IgG1 IRR_H_ 0.55, _95%_CI 0.38–0.80, P = 0.001; IgG3 IRR_M_ 0.69, _95%_CI 0.51–0.92, P = 0.011), and only IgG3 for PvRBP2a (IRR_H_ 0.51 0.36–0.72, P < 0.001) and PvRBP2-P2 (IRR_H_ 0.60, _95%_CI 0.43–0.84, P = 0.002). When combining IgG1 and IgG3 to all PvRBPs in a multivariate model, however, only IgG1 to PvRBP1a (IRR_H_ 0.56, _95%_CI 0.39–0.80, P = 0.001) and PvRBP2b (IRR_M_ 0.64, 0.46–0.90, P = 0.011; IRR_H_ 0.69, _95%_CI 0.48–0.97, P = 0.035) remained associated with reduced risk of vivax-malaria.

### Anti-PvRBP antibody repertoire and risk of vivax-malaria

There was a very strong association between increasing total IgG to the repertoire of PvRBPs, and increase in protection against vivax-malaria. For each increase in one unit of the breadth score (see methods for detailed description), a reduction of approximately 8% in the incidence rate of *P*. *vivax* episodes was observed (IRR 0.92, _95%_CI 0.89–0.96, P < 0.001). Considering the repertoire of IgG1 antibodies, the reduction in risk was of approximately 7% (IRR 0.93, _95%_CI 0.90–0.97, P < 0.001). The effect of the IgG1 repertoire is no longer significant after IgG1 to PvRBP1a (IRR_H_ 0.53, _95%_CI 0.33–0.85, P = 0.008) and PvRBP2b (IRR_M_ 0.63, _95%_CI 0.44–0.89, P = 0.010, IRR_H_ 0.65, _95%_CI 0.42–1.00, P = 0.053) is accounted for, again highlighting importance of these proteins in naturally-acquired protective immunity.

## Discussion

Advances in understanding *P*. *vivax* biology and the acquisition of immunity to this parasite, as well as the development of vaccines against *P*. *vivax* lag much behind what has been achieved for *P*. *falciparum* [14, 26, 27]. This is largely due the lack of a stable *in vitro* culture system for *P*. *vivax* that makes functional studies very challenging.

In this study, we investigated whether six recombinantly expressed members of the PvRBP family are involved in *P*. *vivax* host-cell specificity by testing their erythrocyte-binding preferences. We identified that only PvRBP2b binds solely to reticulocytes. In previous reports, PvRBP1a and PvRBP2c have also been described as reticulocyte-specific binders [8]. Our recombinant PvRBP1a however binds preferentially to normocytes. One explanation for this observation is that C-terminal regions outside of the recombinant construct that is present on native protein governs reticulocyte specificity. In addition, PvRBP1a forms a complex with PvRBP2c [8] in parasites and this complex may be responsible for reticulocyte-binding. Unfortunately, as we were unsuccessful in expressing recombinant PvRBP2c with its binding domain, we were unable to confirm its erythrocyte-binding profile. The molecular mechanisms by which PvRBP2b mediates specific reticulocyte binding, and its reticulocyte-specific receptor are yet to be elucidated.

Since our ability to do functional assays with *P*. *vivax* is constrained due to the lack of *in vitro* culture, we sought to investigate whether antibodies to the 6 PvRBPs are targeted by natural-immunity in a population of young children from PNG. The 6 PvRBPs were recognized differently. The PvRPB2cNB fragment had the lowest immunogenicity of all and antibodies to this fragment did not have a strong association with risk of vivax-malaria. This may be a consequence of the absence of the erythrocyte-binding region. The strongest protective effect was observed for total IgG to PvRBP1a and PvRBP2b. As there is poor cross-reactivity between antibodies targeting the binding regions of different PvRBPs, it is highly likely that these antibodies may have additive or even synergistic effects.

IgG1 and IgG3 were the predominant IgG subclass to PvRBPs in PNG children. Interestingly, the dominant IgG subclass to PvRBP1a was different between children (IgG1) and adults (IgG3). For PvRBP1a and PvRBP2-P2, there was also some early evidence of switching to IgG3 with increasing age. Exposure to malaria parasites, among other antigenic and host characteristics, seems to play a major role in determining the predominant IgG response and for *P*. *falciparum* merozoite antigens, both age and transmission intensity have been previously associated with switching in predominance of the IgG subclass towards IgG3 [28, 29]. Both IgG1 and IgG3 subclasses are cytophilic, T-cell dependent, and bind strongly to Fcγ, mediating phagocyte activation and complement fixation [30, 31], and predominance of IgG1 and/or IgG3, in variable ratios, is common for several *P*. *vivax* [32–34] and *P*. *falciparum* merozoite proteins [35–37]. For the young PNG children included in this study, a reduction in risk of vivax-malaria was observed with IgG3 to PvRBP1a, PvRBP2a and PvRBP2-P2 but, ultimately, it was IgG1 to PvRBP1a and PvRBP2b that showed the strongest associations with protection. Adults also had detectable levels of non-cytophilic IgG2 to most PvRBPs tested however, the significance of this finding remains to be investigated. In *P*. *falciparum* IgG2 antibodies to EBA175 were shown to be short-lived [38], but nevertheless correlated with lower parasitemia. High levels of IgG2 to RESA and MSP2 have also been associated with a lower risk of *P*. *falciparum* infection [39], indicating that although uncommon, IgG2 antibodies might be important for immunity against malaria.

Antibodies to PvRBP2b have also been previously associated with lower parasitaemia in clinical cases [11]. Both proteins seem to be under selective pressure and, in comparison to PvRBP1a, PvRPB2b is less polymorphic and with highly conserved regions, which may be beneficial for vaccine development [12, 13]. Both PvRBP1a and PvRBP2b are less genetically diverse than PvRBP2c [12, 13]. The existence of antigenic diversity in the different PvRBP genes however, has never been investigated. A study with Brazilian samples identified two regions of PvRBP1a (aa 431–748 and 733–1407) as the most immunogenic with predominant IgG1 response, but the relationship between antibodies to the different regions and protection was not explored [18]. Further studies of different regions of both PvRBP1a and PvRBP2b molecules would be important to identify the main epitopes targeted by protective antibodies.

The major limitation of this study is the lack of inclusion of a recombinant protein containing the binding domain of PvRBP2c, precluding both the investigation of the red-cell binding characteristics of PvRBPc and determining relative importance of antibodies targeting red-cell binding fragments of all main PvRBP proteins. Nevertheless, this is the first study where antibody levels to PvRBPs were investigated in samples from a well-designed longitudinal cohort study, which made it possible to adjust for other factors that confound the relationship between antibody acquisition and risk of disease, most importantly the heterogeneity in individual exposure to *P*. *vivax* blood stage infections [20]. The findings of this study provide further insight into *P*. *vivax* host-specificity and naturally-acquired immunity to PvRBPs in children.

While the molecular functions of PvRBPs in *P*. *vivax* invasion are not well understood, the role of the PfRh family, which are homologs of PvRBPs in *P*. *falciparum*, have been well characterized in parasite invasion [3]. Several members of the PfRh family have been implicated in recognition of red blood cells, signaling events or creating a pore in the red blood cell membrane during invasion [40–44]. In particular PfRh5 has been a focus of intense research as a leading blood stage vaccine candidate due to its essential function in *P*. *falciparum* invasion [45, 46]. Apart from gene structure and sequence homology, PvRBP2a and PfRh5 also adopt a similar structural fold within their erythrocyte-binding domains [10, 47, 48]. Using this structural scaffold with varied surface properties PvRBPs and PfRhs are able to mediate alternate receptor engagement. Monoclonal antibodies against PfRh5 results in the strong inhibition of parasite growth across multiple strains and *Aotus nancymaae* monkeys immunized with anti-PfRh5 vaccine are protected against severe infection [42, 49, 50]. It is likely that the erythrocyte-binding domain of PvRBPs will be able to elicit antibodies that have the ability to block *P*. *vivax* invasion.

Our results underline the key role of PvRBPs in parasite–host interactions and highlight their potential as *P*. *vivax* vaccine candidate antigens. Further immuno-epidemiological studies in broader age groups from areas of different transmission intensities, as well as functional studies *in vitro* or in animal models, and a better understanding the molecular function of all PvRBPs, including the PvRBP1a/PvRBP2c complex in *P*. *vivax* invasion are now required to validate and prioritize one or several PvRBPs for development as vaccine candidates.

## Supporting Information

S1 Checklist STROBE checklist. (DOC)

S1 Fig Coomassie-stained SDS-PAGE of recombinant PvRBP2-P2 used in this study. Also indicated is the location of protein molecular mass marker (kDa).
(TIF)

S2 Fig Cumulative distribution function of total IgG levels to 6 PvRBPs in 224 young Papua New Guinean children. (TIF)

S3 Fig IgG subclasses to PvRBPs in Papua New Guinean (PNG) adults and children. Solid lines show antibody levels (arbitrary units) in a dilution series of pooled serum from hyper-immune PNG adults (2-fold, starting 1:50). Dashed lines show median antibody levels observed in 224 young PNG children.
(TIF)

S1 Table Correlation between total and IgG subclasses to PvRBPs in a cohort of 224 young Papua New Guinean children. (XLSX)

S2 Table Associations between IgG and IgG subclasses to 6 RBPs with measures of concurrent and cumulative exposure in a cohort of 224 young Papua New Guinean children. (XLSX)

S3 Table Association between total and IgG subclasses to PvRBPs and protection against clinical malaria (density>500/μL) in a cohort of 224 young Papua New Guinean children. (DOCX)
