# Supplementary material for: Plasmodium vivax Reticulocyte Binding Proteins Are Key Targets of Naturally Acquired Immunity in Young Papua New Guinean Children
Source: PLoS Negl Trop Dis. 2016 Sep 27;10(9):e0005014. doi: 10.1371/journal.pntd.0005014 (PMC5038947; doi:10.1371/journal.pntd.0005014)
Supplement: S1 Checklist — (DOC) [file pntd.0005014.s001.doc]

STROBE Statement—Checklist of items that should be included in reports of ***cohort studies***

|  | Item No | Recommendation |
| --- | --- | --- |
| **Title and abstract** | 1 | (*a*) Use of samples from cohort study cited in abstract |
|  |  | (*b*) Done |
| Introduction | | |
| Background/rationale | 2 | Introduction paragraph 1-3 |
| Objectives | 3 | Introduction paragraph 4 |
| Methods | | |
| Study design | 4 | Section on study population in Methods. Reference to the original Lin et al 2010, PLoS One publication for details |
| Setting | 5 | Section on study population in Methods. Reference to the original Lin et al 2010, PLoS One publication for details |
| Participants | 6 | (Section on study population in Methods. Reference to the original Lin et al 2010, PLoS One publication for details |
|  |  | (*b*) NA |
| Variables | 7 | Sections on study population & statistical analyses in Methods. Reference to the original Lin et al 2010, PLoS One publication for details |
| Data sources/ measurement | 8* | Sections on study population & statistical analyses in Methods. Reference to the original Lin et al 2010, PLoS One publication for details |
| Bias | 9 | Sections on study population & statistical analyses in Methods. Reference to the original Lin et al 2010, PLoS One publication for details |
| Study size | 10 | Defined by size of original study, Lin et al 2010 PLoS one |
| Quantitative variables | 11 | Section on statistical analyses sections in Methods. |
| Statistical methods | 12 | Section on statistical analyses in Methods. Reference to the original Lin et al 2010, PLoS One publication for details |
|  |  | (*b*) Section on statistical analyses n Methods |
|  |  | (*c*) Only children with complete data were included in the analyses – see methods |
|  |  | (*d*) Only children that completed follow-up were included in the analyses |
|  |  | (*e*) ND |
| Results | | |
| Participants | 13* | (a) 224 children in included – see section on study population |
|  |  | (b) see details in Lin et al 2010. PLos One |
|  |  | (c) incl. in Lin et al 2010 |
| Descriptive data | 14* | (a) Section on study population in Methods. Reference to the original Lin et al 2010, PLoS One publication for details |
|  |  | (b) Only children with complete data were included in the analyses – see methods |
|  |  | (c) see details in Lin et al 2010. PLos One |
| Outcome data | 15* | - Clinical episodes of P. vivax Malaria: Described in section on “IgG to Pv RBPs and protection against symptomatic malaria”. Additional details in Lin et al 2010 PLoS one  - Antibody titres: Described throughput the results sections |
| Main results | 16 | (*a*) A complete overview of un-adjusted and adjusted results is given in supplementary tables S1-S3 |
|  |  | (*b*) Category boundaries for antibody measures included into Table 1 |
|  |  | (*c*) ND |
| Other analyses | 17 | Sub-analyses are described in details in the relevant results sections |
| Discussion | | |
| Key results | 18 | Paragraph 2,4,6 of discussion |
| Limitations | 19 | Paragraph 7 plus elsewhere in the discussion where appropriate |
| Interpretation | 20 | Throughout the discussion |
| Generalisability | 21 | Throughout the discussion |
| Other information | | |
| Funding | 22 | Specified in funding section |

*Give information separately for exposed and unexposed groups.

**Note:** An Explanation and Elaboration article discusses each checklist item and gives methodological background and published examples of transparent reporting. The STROBE checklist is best used in conjunction with this article (freely available on the Web sites of PLoS Medicine at http://www.plosmedicine.org/, Annals of Internal Medicine at http://www.annals.org/, and Epidemiology at http://www.epidem.com/). Information on the STROBE Initiative is available at http://www.strobe-statement.org.
