# Supplementary figures and images for: Plasmodium vivax Reticulocyte Binding Proteins Are Key Targets of Naturally Acquired Immunity in Young Papua New Guinean Children
Source: PLoS Negl Trop Dis. 2016 Sep 27;10(9):e0005014. doi: 10.1371/journal.pntd.0005014 (PMC5038947; doi:10.1371/journal.pntd.0005014)

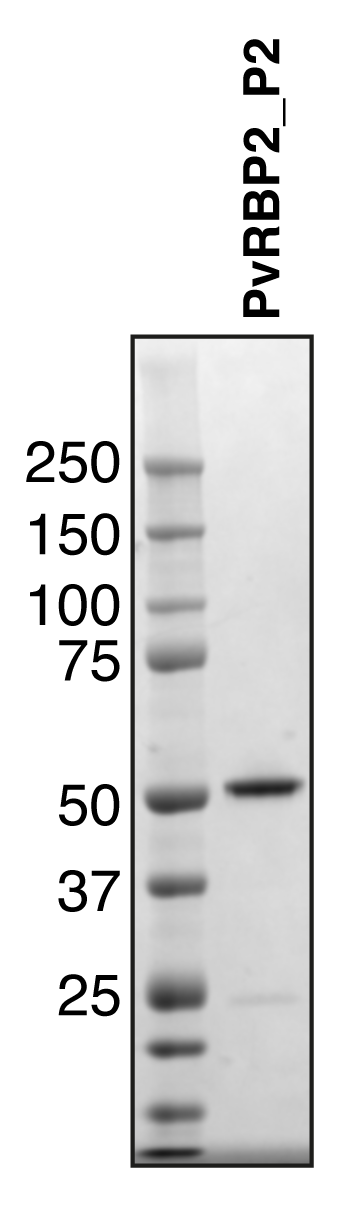

Supplement: S1 Fig — Also indicated is the location of protein molecular mass marker (kDa). (TIF) [file pntd.0005014.s002.tif]

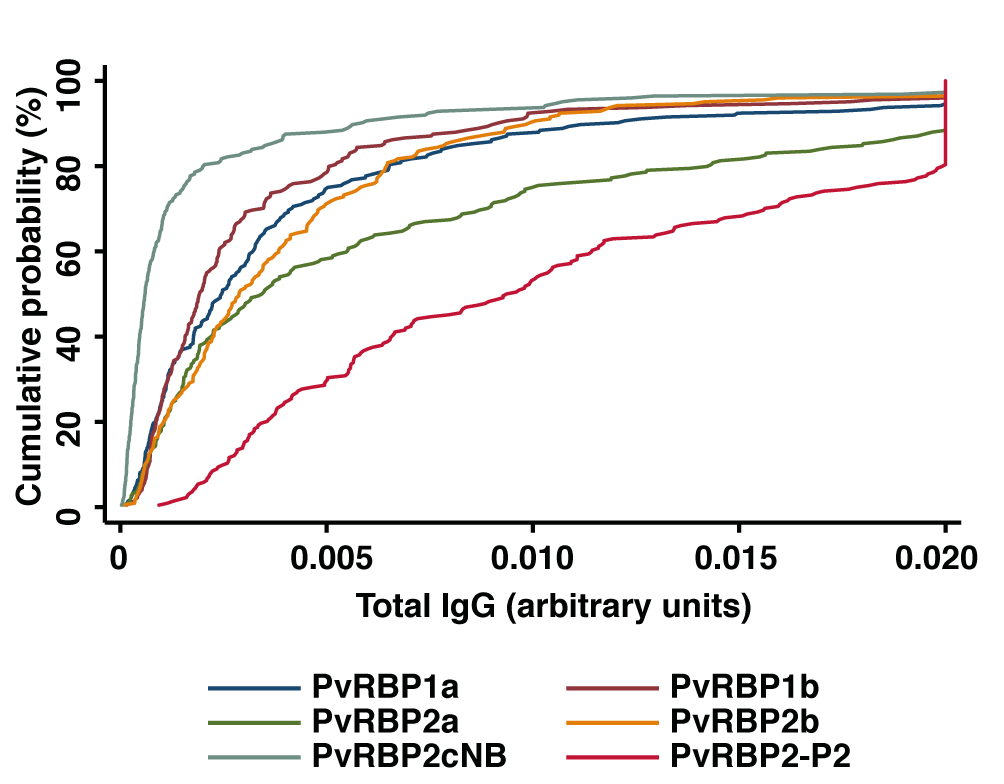

Supplement: S2 Fig — (TIF) [file pntd.0005014.s003.tif]

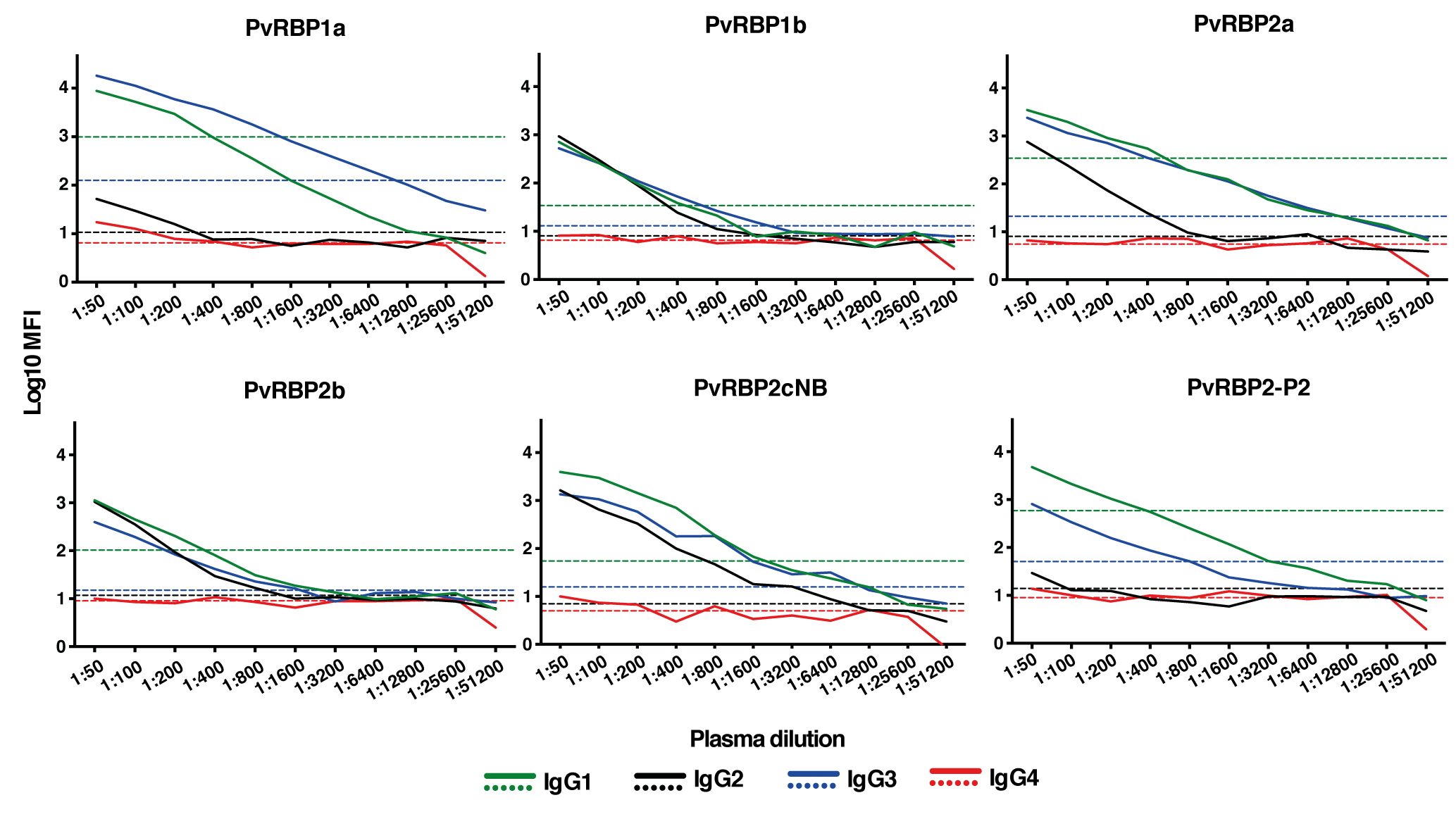

Supplement: S3 Fig — Solid lines show antibody levels (arbitrary units) in a dilution series of pooled serum from hyper-immune PNG adults (2-fold, starting 1:50). Dashed lines show median antibody levels observed in 224 young PNG children. (TIF) [file pntd.0005014.s004.tif]
