# Supplementary material for: Plasmodium vivax Reticulocyte Binding Proteins Are Key Targets of Naturally Acquired Immunity in Young Papua New Guinean Children
Source: PLoS Negl Trop Dis. 2016 Sep 27;10(9):e0005014. doi: 10.1371/journal.pntd.0005014 (PMC5038947; doi:10.1371/journal.pntd.0005014)
Supplement: S3 Table — (DOCX) [file pntd.0005014.s007.docx]

**Table S3:** Association between total and IgG subclasses to PvRBPs and protection against clinical malaria (density>500/ul) in a cohort of 224 young Papua New Guinean children.

| **Antigen** | **Antibody** | **uIRR** | **95%CI** | | ***p-*value** | **aIRR** | **95%CI** | | ***p-*value** |
| --- | --- | --- | --- | --- | --- | --- | --- | --- | --- |
|  | Total IgG M | 0.59 | 0.43 | 0.82 | **0.001** | 0.68 | 0.51 | 0.90 | **0.008** |
|  | Total IgG H | 0.49 | 0.33 | 0.71 | **<0.001** | 0.53 | 0.38 | 0.74 | **<0.001** |
| PvRBP1a | IgG1 M | 0.67 | 0.49 | 0.93 | **0.018** | 0.76 | 0.57 | 1.03 | 0.075 |
|  | IgG1 H | 0.46 | 0.32 | 0.68 | **<0.001** | 0.48 | 0.35 | 0.67 | **<0.001** |
|  | IgG3 M | 0.62 | 0.44 | 0.88 | **0.008** | 0.67 | 0.50 | 0.91 | **0.011** |
|  | IgG3 H | 0.44 | 0.31 | 0.63 | **<0.001** | 0.51 | 0.36 | 0.72 | **<0.001** |
|  | Total IgG M | 0.90 | 0.64 | 1.27 | 0.537 | 0.81 | 0.62 | 1.08 | 0.147 |
|  | Total IgG H | 0.54 | 0.37 | 0.78 | **0.001** | 0.52 | 0.37 | 0.73 | **<0.001** |
| PvRBP1b | IgG1 M | 0.67 | 0.48 | 0.93 | **0.019** | 0.86 | 0.62 | 1.18 | 0.350 |
|  | IgG1 H | 0.49 | 0.34 | 0.70 | **<0.001** | 0.52 | 0.37 | 0.74 | **<0.001** |
|  | Total IgG M | 0.95 | 0.67 | 1.34 | 0.759 | 0.87 | 0.65 | 1.16 | 0.335 |
|  | Total IgG H | 0.69 | 0.47 | 1.01 | 0.056 | 0.61 | 0.44 | 0.84 | **0.003** |
|  | IgG1 M | 0.87 | 0.61 | 1.24 | 0.446 | 0.88 | 0.66 | 1.18 | 0.400 |
| PvRBP2a | IgG1 H | 0.80 | 0.55 | 1.17 | 0.251 | 0.66 | 0.48 | 0.91 | **0.010** |
|  | IgG3 M | 0.75 | 0.53 | 1.05 | 0.089 | 0.76 | 0.57 | 1.01 | 0.055 |
|  | IgG3 H | 0.46 | 0.32 | 0.67 | **<0.001** | 0.51 | 0.36 | 0.72 | **<0.001** |
|  | Total IgG M | 0.58 | 0.41 | 0.81 | **0.002** | 0.69 | 0.51 | 0.93 | **0.016** |
|  | Total IgG H | 0.45 | 0.31 | 0.65 | **<0.001** | 0.53 | 0.38 | 0.76 | **<0.001** |
| PvRBP2b | IgG1 M | 0.53 | 0.37 | 0.75 | **<0.001** | 0.59 | 0.43 | 0.81 | **0.001** |
|  | IgG1 H | 0.49 | 0.35 | 0.69 | **<0.001** | 0.57 | 0.40 | 0.80 | **0.001** |
|  | Total IgG M | 0.77 | 0.55 | 1.08 | 0.131 | 0.85 | 0.63 | 1.14 | 0.266 |
|  | Total IgG H | 0.45 | 0.31 | 0.64 | **<0.001** | 0.58 | 0.41 | 0.82 | **0.002** |
| PvRBP2cNB | IgG1 M | 1.03 | 0.73 | 1.47 | 0.848 | 1.05 | 0.79 | 1.40 | 0.739 |
|  | IgG1 H | 0.66 | 0.46 | 0.97 | **0.033** | 0.77 | 0.55 | 1.06 | 0.111 |
|  | Total IgG M | 0.59 | 0.41 | 0.84 | **0.003** | 0.65 | 0.47 | 0.89 | **0.008** |
|  | Total IgG H | 0.63 | 0.44 | 0.91 | **0.013** | 0.66 | 0.47 | 0.91 | **0.013** |
|  | IgG1 M | 0.82 | 0.57 | 1.19 | 0.295 | 0.89 | 0.65 | 1.21 | 0.452 |
| PvRBP2-P2 | IgG1 H | 0.80 | 0.56 | 1.15 | 0.227 | 0.86 | 0.62 | 1.20 | 0.371 |
|  | IgG3 M | 0.78 | 0.56 | 1.10 | 0.156 | 0.94 | 0.69 | 1.27 | 0.667 |
|  | IgG3 H | 0.51 | 0.35 | 0.73 | **<0.001** | 0.60 | 0.43 | 0.84 | **0.002** |

Abbreviations: M=Medium antibody levels; H=High antibody levels.

uIRR: Unadjusted incidence rate ratio.

aIRR: Adjusted incidence rate ratio. Adjustments were made for individual differences in exposure (molFOB), age, season and village.

uIRR, aIRR and P-values from GEE models. P<0.05 was deemed significant.
